# Analysis of the Changes in Volatile Components During the Processing of Enshi Yulu Tea

**DOI:** 10.3390/foods13233968

**Published:** 2024-12-09

**Authors:** Anhui Gui, Fei Ye, Jinjin Xue, Shengpeng Wang, Panpan Liu, Xueping Wang, Jing Teng, Lin Feng, Jun Xiang, Pengcheng Zheng, Shiwei Gao

**Affiliations:** 1 Key Laboratory of Tea Resources Comprehensive Utilization (Co-Construction by Ministry and Province), Ministry of Agriculture and Rural Affairs, Fruit and Tea Research Institute, Hubei Academy of Agricultural Sciences, Wuhan 430064, China; guianhui@hbaas.com (A.G.); yf421@163.com (F.Y.); xuejinjin911@163.com (J.X.); wwsspp0426@163.com (S.W.); liuppitea@163.com (P.L.); wangxueping79-79@163.com (X.W.); jobbase@163.com (J.T.); fenglin@hbaas.com (L.F.); zpct15@163.com (P.Z.); 2 Enshi Tujia and Miao Autonomous Prefecture Academy of Agricultural Sciences, Enshi 445002, China; xiangjun@tricaas.com

**Keywords:** Enshi Yulu tea, steamed green tea, volatile components, variety

## Abstract

Volatile constituents are critical to the flavor of tea, but the changes in Enshi Yulu tea during the processing have not been clearly understood. Using headspace solid phase microextraction combined with gas chromatography-mass spectrometry (HS-SPME/GC-MS) techniques, we analyze the aroma components of Enshi Yulu tea and changes in them during the processing stages. In total, 242 volatile compounds were identified. From fresh leaves to the shaping process in tea production, there are significant decreases in overall aroma substances, followed by increases after drying. Linalool is the dominant aroma component in Enshi Yulu tea, with a proportion of 12.35%, followed by compounds such as geraniol (7.41%), 2,6-dimethyl-5-heptene (6.93%), phenylmethanol (5.98%), isobutyl acetate (4.16%), hexan-1-ol (3.95%), 2-phenylacetaldehyde (3.80%), and oct-1-ene-3-ol (3.34%). The number of differential volatile components varied by production stage, with 20 up- and 139 down-regulated after steaming, 24 down-regulated after rolling, 60 up- and 51 down-regulated after shaping, and 68 up- and 13 down-regulated after drying. Most variation in expression occurred because of steaming, and the least during the rolling stage. PLS-DA analysis revealed significant differences in aroma components throughout processing and the identification of 100 compounds with higher relative contents, with five distinct change trends. Phenylmethanol, phenylacetaldehyde, *(2E)*-non-2-enal, oct-1-ene-3-ol, and *cis*-3-hexenyl hexanoate could exert a profound influence on the overall aroma quality of Enshi Yulu tea during processing. The results offer a scientific foundation and valuable insights for understanding the volatile composition of Enshi Yulu tea and its changes during the processing.

## 1. Introduction

Tea, processed from the tender shoots of tea plants (*Camellia sinensis* (L) O. Kuntze), is one of the most commonly consumed aromatic beverages, next to water, in the world. In China, tea production encompasses six distinct types, including green, black, oo-long, white, yellow, and dark teas. These classifications are based on their manufacturing processes and sensory attributes [1]. Green tea, an unfermented variety of tea, is widely renowned for its exquisite flavor and potential health benefits. The fixation process holds paramount importance in the production of green tea, as it involves subjecting fresh leaves to high-temperature treatment to inhibit enzyme activity [2]. The fundamental manufacturing processes of green tea encompass fixing, rolling, shaping, and drying [1]. In the realm of Chinese green tea, pan-frying is the predominant fixation method, while Japanese green teas typically undergo steaming fixation [3]. Enshi Yulu tea, which dates back to the Tang Dynasty, distinguishes itself as the sole representative of Chinese green tea that employs steaming fixation. It has significantly different aroma components.

The aroma of tea is important in quality assessment and exerts a direct influence on the tea’s overall value. The findings of multiple studies have consistently demonstrated that tea aroma consists of two components: the inherent fragrance derived from fresh leaves and the aroma produced during processing [4]. Thus, the volatile compounds in teas are greatly influenced by the tea cultivar [5], production season [6], altitude [7], shading treatment [8], manuring [9], and processing method [10]. The aroma of Enshi Yulu tea has been the subject of scholarly research. Gong et al. found that *cis*-3-hexenol and *cis*-3-hexenyl caproate are some of the most prominent compounds in Enshi Yulu tea. Enshi Yulu tea’s fragrance differs from pan-fried green teas [11]. In other studies, some aroma compounds, such as *cis*-3-hexenyl butyrate and *cis*-3-hexenyl caprylate, are also unique to this tea [12]. Shi et al. found that the volatile compounds in steamed green tea differed significantly from those in pan-fried green tea. Compounds with higher relative contents in Enshi Yulu tea include linalool, nonaldehyde, limonene, phenylmethanol, and other compounds [3]. The aforementioned studies primarily focus on Enshi Yulu tea, with no consideration given to the changes in aroma compounds during the processing stage.

Although many studies have demonstrated significant changes in the aroma composition of green tea during processing, particularly during the fixation and drying stages [13,14,15], these studies have focused on pan-fried as opposed to steamed green tea processing. Due to its special flavor, which differs from that of pan-fried green tea, Enshi Yulu tea has recently attracted the attention of tea consumers and researchers. Therefore, to remedy this deficiency, we use headspace solid phase microextraction combined with gas chromatography-mass spectrometry (HS-SPME/GC-MS) techniques to investigate changes in aroma components during the processing of Enshi Yulu tea. The results of our study provide novel insights into the formation mechanism of the qualities of steamed green tea.

## 2. Materials and Methods

### 2.1. Chemicals and Reagents

Deionized water was obtained using a Milli-Q water purification system (Millipore, Billerica, MA, USA). NaCl was obtained from the J&K Scientific Corporation (Beijing, China), and hexan-3-one was obtained from the Sigma-Aldrich Corporation (Shanghai, China).

### 2.2. Tea Samples

Approximately 100 kg of fresh tea leaves of Clonal Longjing 43 cultivar (*Camellia sinensis*) were obtained from the tea gardens of Jinfeng Tea Co., Ltd. (Enshi, China) on 30 March 2022. Fresh tea leaves included one bud with two leaves. After naturally withering for approximately 4 h at 20–25 °C on bamboo sieves, the fresh leaves were continuously fixed with steam (3.8–4.0 MPa) and hot air (520–550 °C) for 40–50 s using a steam-hot-air fixation machine (6CZQRC-300, Sichuan Dengyao Machinery Equipment Co., Ltd., Chengdu, China). Fixed leaves were then dehydrated for 40–50 s at 150–180 °C prior to rolling for 40 min using a 6CR-55 rolling machine (Hubei Tianchi Machines Co., Ltd., Yichang, China). Rolled leaves were then shaped in a three-step process: (a) they were first dried to approximately 45% moisture content at 100–120 °C; (b) shaped using a shaping machine (6CL-12/80, Sichuan Dengyao Machinery Equipment Co., Ltd., Chengdu, China) at 90–110 °C for approximately 30 min; and (c) a second shaping using a fine shaping machine (60K-S, Zhejiang Kawasaki Tea Machinery Co., Ltd., Hangzhou, China) at 90 °C for approximately 45 min to 25% moisture content. Finally, shaped leaves were dried at 90–100 °C for 20 min until the moisture content was <6%.

After each process, samples of fresh tea leaves (FTL), steamed tea leaves (STL), rolled tea leaves (RTL), shaped tea leaves (SPTL), and dried tea leaves (DTL) were collected, immediately frozen in liquid nitrogen, then freeze-dried (FD 5–10; SIM International Co., Ltd., Los Angeles, California, USA) at −50 °C for 48 h. Three replicate samples were collected simultaneously from different points during a single process. Additionally, equal amounts of each sample were mixed for quality control. Samples were stored at −20 °C until further analysis.

### 2.3. Volatile Component Analysis

Volatile components were extracted using a Head Space Solid Phase Microextraction (HS-SPME) method. The sample was removed from the −20 °C refrigerator and transferred to a drying dish. After reaching room temperature, approximately 0.5 g of the sample was weighed and placed into a 20 mL headspace bottle, to which 6 mL of saturated NaCl solution and 10 μL of hexan-3-one (50 μg/mL) as an internal standard. At constant temperature (60 °C), after oscillating for 5 min, the DVB/CWR/PDMS extraction head was inserted for 15 min to absorb volatile components. After extraction, desorption at 250 °C for 5 min was performed before performing Gas Chromatography-Mass Spectrometry (GC-MS) analysis on each sample five times. To ensure instrument stability, for every 10 samples to monitor the repeatability of the analytical process, quality control samples were tested three times (Figures S1 and S2).

GC–MS analysis was carried out using a GC system (8890, Agilent Technologies, Santa Clara, CA, USA) coupled with an MS detector (7000D, Agilent Technologies, Santa Clara, CA, USA). GC-MS chromatographic conditions included: DB-5MS capillary column (30 m × 0.25 mm × 0.25 μm, Agilent J&W Scientific, Folsom, CA, USA), high purity helium carrier gas (purity not less than 99.999%) at 1.2 mL/min constant flow rate; inlet temperature 250 °C, without split injection, with solvent delay set at 3.5 min; programmed temperature rise starting at 40 °C for 3.5 min followed by an increase of 10 °C/min to 100 °C, 7 °C/min to 180 °C, and 25 °C/min to 280 °C, with a hold time of 5 min. GC-MS mass spectrometry conditions included: electron bombardment ion source (EI), ion source temperature (230 °C), quadrupole bar temperature (150 °C), mass spectrum interface temperature (280 °C), electron energy (−70 eV); scanning mode selected ion detection mode (SIM); and qualitative and quantitative ion precision scanning (GB 23200.8—2016).

The analysis of volatile compounds was performed with the assistance of MetWare (http://www.metware.cn/, accessed on 23 September 2022) corporation (Wuhan, China), a professional institution for analysis. The volatile compounds were characterized by matching their mass spectra against the data system library (MetWare Gas Chromatography, MWGC) and linear retention index. For each compound, one quantitative ion and two or three qualitative ions were carefully chosen. Each group of ions was sequentially detected during their respective time segments based on their elution order. To confirm the identification, the observed retention time was compared with the standard reference, and the presence of all selected ions in the sample spectrum after background subtraction was verified [16]. For improved quantitative accuracy, specific quantitative ions were chosen for integration and calibration. The internal standard, hexan-3-one (10 µL, 50 µg/mL), was employed, and the relative content of each volatile was computed as follows:

$$C_{j}=\frac{A_{j}\times V_{i}\times C_{i}}{A_{i}\times m_{s}}$$

where *C_j_* denotes the mass concentration of each compound (µg/kg); *A_j_* and *A_i_* represent the chromatographic peak areas of the compound and internal standard, respectively; *V_i_* means the volume of internal standard (10 µL); *C_i_* means the concentration of the internal standard (50 µg/mL); *m_s_* means the mass of sample powder (kg).

### 2.4. Data Analysis

Partial least squares discriminant analysis (PLS-DA) was performed using Simca-P 13.0 software (Umetrics AB, Umea, Sweden) after the data were UV-scaled. A heat-map was generated using MultiExperiment Viewer 4.8.1 (Oracle Corporation, Redwood, CA, USA) after the data were UV-scaled. The significance of the differences in the pairwise comparisons and the whole comparison was calculated via one-way analysis of variance (ANOVA, New Providence, NJ, USA) and a Tukey s-b(K) test using PASW Statistics software (Version 18.0, Chicago, IL, USA), respectively.

## 3. Results and Discussion

### 3.1. Profile Analysis of Volatile Components

**Figure 1:**
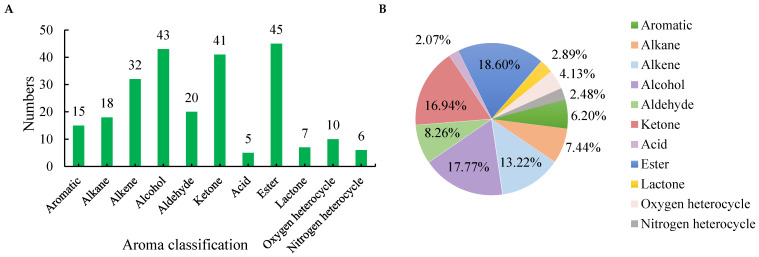
Numbers (**A**) and percentage (**B**) of different aroma classification in Enshi Yulu tea.

After peak matching and pollutant removal, 242 reliable volatile components were identified (Table S1). Based on chemical structure, these components were categorized into 11 classes (Figure 1A, Table S1), among which the largest was esters (45 counts, 18.60%), followed by alcohols (43, 17.77%), ketones (41, 16.94%), alkenes (32, 13.22%), and aldehydes (20, 8.26%), which collectively accounted for >8%. There were over 10 alkanes (18, 7.44%), aromatic (15, 6.20%), and oxygen heterocycles (10, 4.13%); quantities of lactones (7, 2.89%), nitrogen heterocycles (6, 2.48%), and acids (5, 2.07%) were relatively low (Figure 1B). Consistent with these findings, further validating test-result reliability, is that higher proportions of esters, alcohols, and ketones have been reported as aroma components in steamed green tea [3,17].

**Figure 2:**
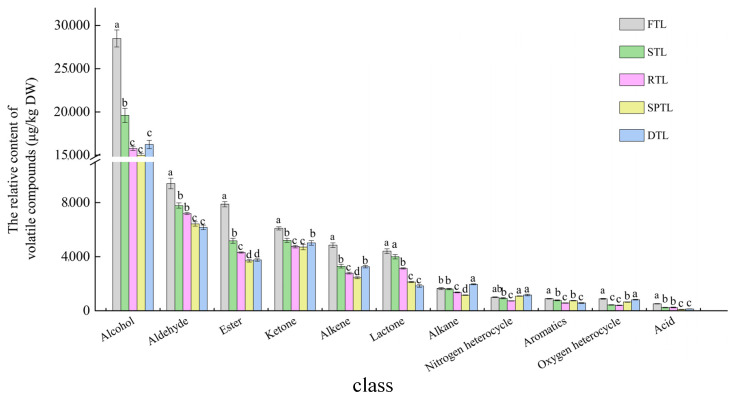
The relative content (μg/kg) of different classes of volatiles in the processing stages of Enshi Yulu tea. FTL, fresh tea leaves; STL, steamed tea leaves; RTL, rolled tea leaves; SPTL, shaped tea leaves; DTL, dried tea leaves. All values are shown as mean ± SD. Distinct letters (a–d) within the identical class signify significant variations among the processing stage of Enshi Yulu tea (*p* < 0.05).

Levels of various volatile compounds, including alcohols, aldehydes, ketones, esters, lactones, aromatic hydrocarbons, alkanes, olefins, and acids, significantly decreased from FTL to RTL stages, particularly during the steaming process (Figure 2). During the shaping process, these compounds declined further while volatiles of oxyheterocycles and nitrogen heterocycles increased (Figure 2). During drying, there was a significant increase in alcohol, ketone, alkane, acid, oxyheterocycle, and nitrogen heterocycle contents and a notable decrease in aromatic hydrocarbon contents; other compounds remained relatively unchanged (Figure 2). These findings align with research on changes in aroma components during the drying process of roasted green tea [14]. Overall, the total aroma gradually decreased from FTL to shaping but increased after drying.

The aroma of tea is related to the relative proportions of different volatiles. Whether fresh leaves or finished tea, alcohol volatiles have the highest relative content (approximately 40%), followed by aldehydes, esters, ketones, and others (Table 1). Shi et al. found that the steamed green tea has the highest proportion of alcohols (50.02%), followed by esters (14.32%) [3], possibly because there are high concentrations of alcohols, aldehydes, ketones, and other aromatic components in fresh tea leaves [18]. During processing from FTL to the DTL, the proportional contributions of aromatic hydrocarbons, alkanes, aldehydes, ketones, and nitrogen heterocycles trend upwards, and olefins, alcohols, acids, esters, and lactones trend downwards. After completion of the drying process, levels of aromatic hydrocarbons, aldehydes, and lactones decreased significantly while those of alkane compounds, acids, and oxygen heterocycles increased; levels of remaining compounds changed little. A substantial increase in oxygen and nitrogen acyclic groups during shaping and drying processes can be attributed to an enhanced Maillard reaction during these steps [19,20].

The influence of volatile components on aroma quality is often closely related to their contents. Of individual aroma components in the DTL stage, the proportional contributions of the top 40 components (with proportion ≥ 0.44%) are presented in Table 2. Of these aroma components, linalool, geraniol, phenylmethanol, oct-1-en-3-ol, benzaldehyde, 2-phenylacetaldehyde, β-ionone, *(2E)*-non-2-enal, *trans*-linalool oxide (furanoid), *cis*-3-hexenyl hexanoate, hexanal, *(Z)*-3-hexen-1-ol, naphthalene, *trans*-linalool oxide (pyranoid), and hexan-1-ol are compounds with higher relative contents in green tea [3,21,22,23,24,25,26,27], and *cis*-3-hexenyl valerate is a compound with higher relative content in Yunnan black tea [28]. Most of these components present pleasant aromas. For example, linalool, geraniol, phenylmethanol, benzaldehyde, 2-phenylacetaldehyde, β-ionone, and *cis*-3-hexenyl valerate have pleasant fragrances of flowers, fruits, and sweetness (Table 2). Those such as hexan-1-ol, oct-1-en-3-ol, *(Z)*-3-hexen-1-ol, and *(2E)*-non-2-enal are derived from fatty acid degradation [4,18] and are fragrant at low concentrations. At high concentrations, they are characterized by grassy smells [26,29,30] (Table 2). Naphthalene presents with an unpleasant reagent taste (Table 2). These aroma components are the potential basis of the fresh orchid aroma of Enshi Yulu tea.

### 3.2. Effects of Processing Steps on Volatile Components

**Figure 3:**
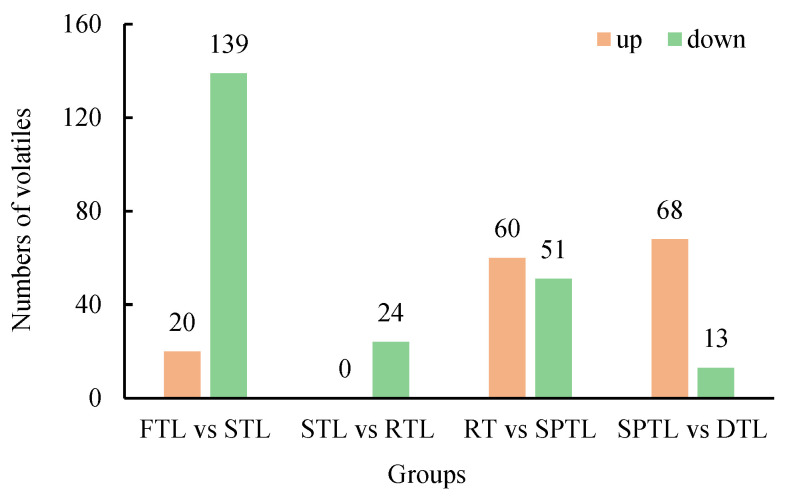
Numbers of differential volatiles in the processing stages Enshi Yulu tea. FTL, fresh tea leaves; STL, steamed tea leaves; RTL, rolled tea leaves; SPTL, shaped tea leaves; DTL, dried tea leaves.

To better understand the impact of processing steps on volatile components, we screened differential compounds (*p* < 0.01) with a fold change ≥ 1.50 (up-regulated) or ≤0.67 (down-regulated) at each stage. The most pronounced variations in aroma components occurred during the steaming process (Figure 3, Table S2), where the contents of 159 volatile components changed significantly (20 up-regulated, 139 down-regulated). Only 24 distinct compounds differed during the rolling process, and all were down-regulated. Following shaping, there were notable changes in 106 volatile components, of which 60 were up-regulated and 51 were down-regulated. Drying led to significant modifications in 76 volatile components, of which 67 were up-regulated, and 13 were down-regulated. The high number of differential volatile components after green tea steaming further confirms the importance of this step in determining green tea quality. Shaping and drying processes also exert considerable influence on the aroma of Enshi Yulu tea.

**Figure 4:**
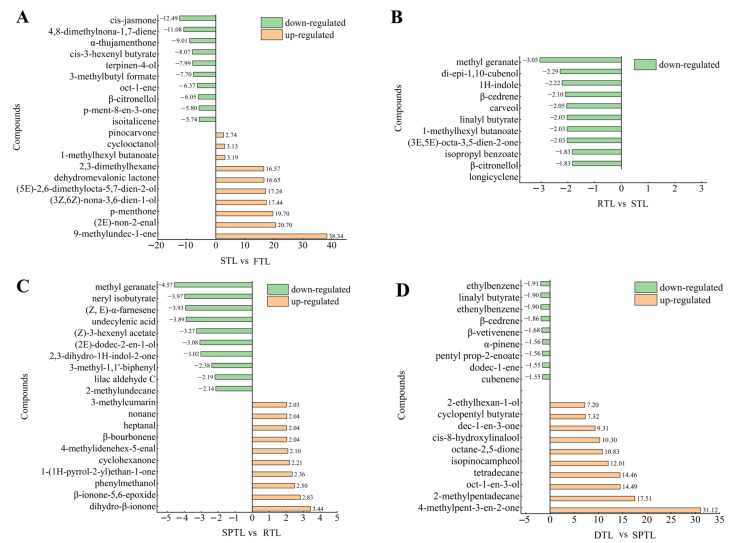
Bar chart of differential compounds in the processing stages of Enshi Yulu tea: (**A**) STL vs. FTL, (**B**) RTL vs. STL, (**C**) SPTL vs. RTL, and (**D**) DTL vs. SPTL. Orange: up-regulated compounds; Green: down-regulated compounds. The number above the column means fold change. FTL, fresh tea leaves; STL, steamed tea leaves; RTL, rolled tea leaves; SPTL, shaped tea leaves; DTL, dried tea leaves.

To better understand the influence of the way that processing methods affect volatile components, we performed extensive screening and comparison of differential compounds (TDCs) that most significantly varied at each processing stage (Figure 4).

After steaming, up-regulated TDCs comprised compounds such as 9-methyl-1-undecene, *(2E)*-non-2-enal, *p*-menthone, *(3Z,6Z)*-nona-3,6-dien-1-ol, and *(5E)*-2,6-dimethylocta-5,7-dien-2-ol. These compounds exhibited a wide range of multiplicities ranging from 2.74 to 38.34 (Figure 4A). Conversely, down-regulated TDCs included *cis*-jasmone, 4,8-dimethyl-1,7-nonadiene, α-thujamenthone, *cis*-3-hexenyl butyrate, terpinen-4-ol, 3-methylbutyl formate, oct-1-ene, β-citronellol, *p*-mentha-8-en-3-one, and isoitalicene, with a decline rate up to approximately 12.49 times (*cis*-jasmone) (Figure 4A). During steaming, the degradation of fatty acids and carotenoids into aroma components is facilitated by both thermodynamic action at high temperatures and enzymatic activity prior to enzyme inactivation [36,37]. Moreover, sugars and amino acids undergo the Maillard reaction, leading to the generation of heterocyclic aroma components [20]. However, elevated temperatures can also cause substantial volatilization of aroma components. Additionally, during the fixing process, alcohol-based aroma components can combine with glucose and primrose sugar to form glycosidically bound volatiles [38], which inevitably reduces the contents of alcohol-based aroma components. Variation in molecular weight and polarity of aroma components produces different volatility levels. During the steaming process, the volatilization rate may exceed the replenishment rate for most aroma components, with few exhibiting the opposite trend. Consequently, most aroma components decrease considerably following steaming, while a few, such as *(2E)*-non-2-enal and *(3Z,6Z)*-nona-3,6-dien-1-ol, increase.

After rolling, including methyl geranate, di-epi-1, 10-cubenol, 1H-indole, β-cedrene, carveol, linalyl butyrate, and *(3E,5E)*-octa-3,5-dien-2-one were down-regulated (Figure 4B). Compared with the steaming process, the range of variation in volatiles after rolling was lower—an observation consistent with changes in the total amount of aroma components (Figure 2). It is customary to allow Enshui Tea to cool to ambient temperature before rolling it to preserve its green color and inhibit enzyme activity. Consequently, during the rolling process, enzymatic reactions cannot generate aroma components, and thermodynamic reactions also fail to produce these compounds; only volatilization of existing aroma components occurs, causing a slight reduction in their abundance. Aroma components typically have low volatility at ambient temperature, leading to a minor decrease.

After shaping, the up-regulated TDCs included dihydro-β-ionone, β-ionone-5,6-epoxide, phenylmethanol, 1-(1H-pyrrol-2-yl)-ethanone, cyclohexanone and others. Down-regulated TDCs included methyl geranate, neryl isobutyrate, *(Z, E)*-α-farnesene, undecylenic acid, *(Z)*-3-hexenyl acetate, *(2E)*-dodec-2-en-1-ol, 2,3-dihydro-1H-indol-2-one, lilac aldehyde C and others. (Figure 4C). Down-regulated TDCs were significantly higher than the up-regulated TDCs, irrespective of the extent or magnitude of change, suggesting that the shaping process exerted a comparable impact to steaming on the aroma of Enshi Yulu tea During production, the shaping process of Enshi Yulu tea usually occurs at approximately 100 °C—a temperature at which many aroma components volatilize, leading to a decrease in their contents (Figure 4C). However, high temperatures can promote the degradation of carotenoids into β-ionone, geranylacetone, 5,6-epoxy-β-ionone, and other aroma components [36]. Sugars and amino acids also generate heterocyclic aroma components through the Maillard reaction [20]. In up-regulated TDCs, β-ionone-5,6-epoxide, and dihydro β-ionone are volatile products of carotenoid degradation [39]. 1-(1H-pyrrol-2-yl)-ethanone is a product of the Maillard reaction [20].

After drying, the up-regulated TDCs include 4-methyl-3-penten-2-one, 2-methyl-pentadecane, oct-1-en-3-ol, tetradecane, isopinocampheol, octane-2,5-dione, *cis*-8-hydroxylinalool, dec-1-en-3-one, and cyclopentyl butyrate. Down-regulated TDCs comprise ethylbenzene, linalyl butyrate, ethenylbenzene, and β-cedrene (Figure 4D). The magnitude of change in down-regulated TDCs is greater than up-regulated ones in both quantity and change factor—an observation consistent with a significant increase in total aroma after drying (Figure 2). Despite similar high-temperature conditions in the shaping and drying processes under identical processing technology for Enshi Yulu tea production, the compounds’ alteration trends after drying are essentially opposite to those observed during the shaping process, with evident differences in TDCs. These findings indicate that the effects of shaping and drying on aroma formation in Enshi Yulu tea differ. Although both processes occur subsequent to the fixing step and involve only thermodynamic actions, the water content differs between them: during shaping, the tea’s moisture content must be reduced from approximately 65% to 25%, and during the drying process, it is further decreased to <6%. The moisture content of tea leaves is greater during shaping, favoring moist heat reactions, and lower during drying, which may promote dry heat reactions instead. Water plays an important role in product formation through the Maillard reaction within the glucose-glycine/proline/lysine model system [40] and degradation of carotenoids leading to aroma component generation [36,41]. Therefore, differences in moisture content may explain variations in aroma formation in the shaping and drying stages of Enshi Yulu tea.

### 3.3. Evolution of Differential Volatiles Throughout Enshi Yulu Tea Production

**Figure 5:**
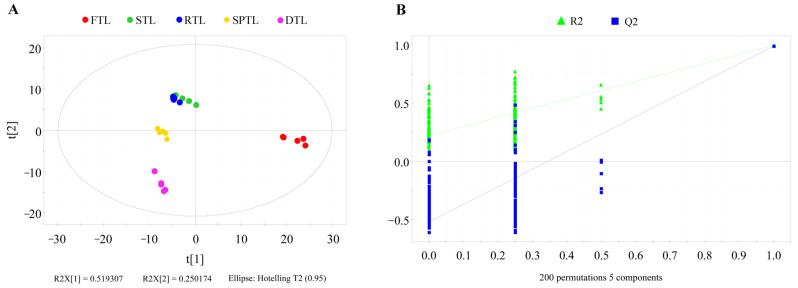
PLS-DA analysis of volatile compounds in the processing stage of Enshi Yulu tea. FTL, fresh tea leaves; STL, steamed tea leaves; RTL, rolled tea leaves; SPTL, shaped tea leaves; DTL, dried tea leaves. (**A**), score plot (R^2^X = 0.926, R^2^Y = 0.981, Q^2^ = 0.951); (**B**), Cross-validation test (R^2^ = 0.222; Q^2^ = −0.523).

To enhance the differentiation of aroma components during the processing stages, we used partial least squares discriminant analysis (PLS-DA). The model exhibited excellent fitting and predictive capabilities (R^2^X = 0.926, R^2^Y = 0.981, Q^2^ = 0.951) (Figure 5A). Five sample groups were effectively distinguished based on their origin, indicating significant variations in volatile composition existed. The greatest dissimilarity was between FTL and STL samples, suggesting that the steaming process exerted a profound influence on the formation of Enshi Yulu tea’s aroma. The drying process also played an important role in aroma development, while the effect of rolling was relatively minor. These findings are consistent with research on how processing techniques affect the aroma of roasted green teas [14]. To assess model robustness, we performed a permutation test with 200 repetitions for cross-validation; overfitting did not occur (Figure 5B: R^2^ = 0.222; Q^2^ = −0.523).

**Figure 6:**
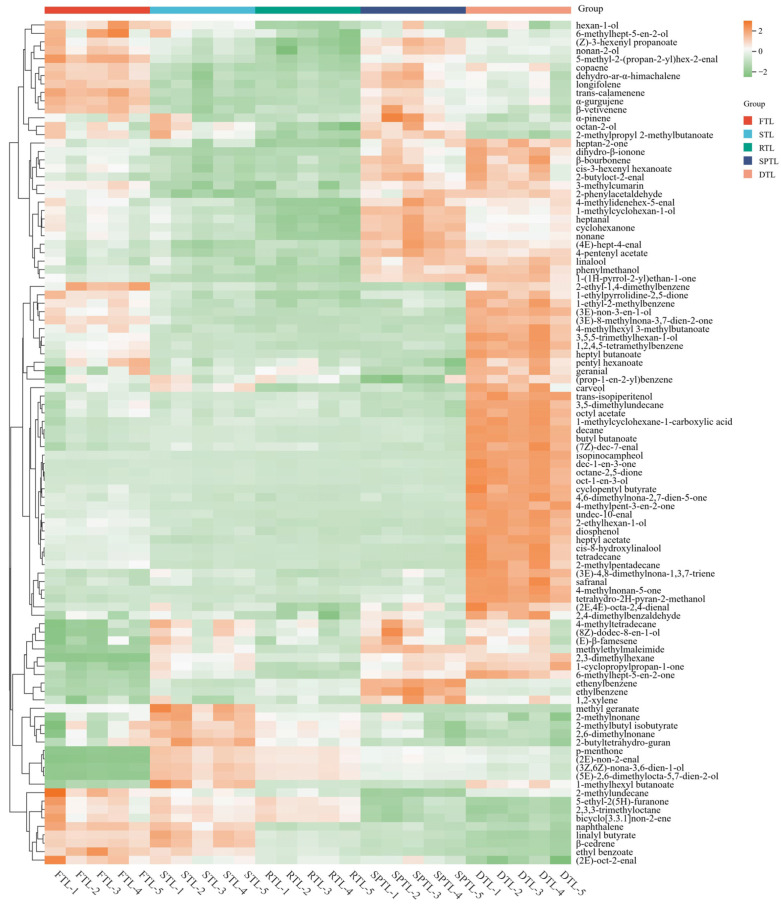
Heat map of possible key-differential volatile components during the processing of Enshi Yulu tea. FTL, fresh tea leaves; STL, steamed tea leaves; RTL, rolled tea leaves; SPTL, shaped tea leaves; DTL, dried tea leaves.

To identify potential volatile component markers for the processing stages of Enshi Yulu tea, we used their VIP (Variable Importance in Projection) values in the PLS-DA model, combined with non-parametric testing (Kruskal–Wallis). A total of 100 volatile components exhibited VIP > 1 (Table S1). These volatile components varied significantly throughout the processing stages, with a distinct inflection point after steaming and drying (Figure 6). These volatile components displayed five main trends: (1) compounds such as 5-ethyl-2(5H)-furanone, 2,3,3-trimethyloctane, naphthalene, linalyl butyrate, ethyl benzoate, *(2E)*-oct-2-enal that decreased slightly after rolling and significantly decreased during shaping and drying processes. (2) Compounds such as *(2E)*-non-2-enal, methyl geranate, *(3Z,6Z)*-nona-3,6-dien-1-ol, 2-methylnonane, 2-methylbutyl isobutyrate, 2,6-dimethylnonane, and *p*-menthone that increased significantly after steaming and gradually decreased from the rolling to drying processes. (3) Compounds such as octyl acetate, 4-methyl-3-penten-2-one, octane-2,5-dione, oct-1-en-3-ol, and safranal that did not differ significantly in steaming, rolling, and shaping processes, but significantly increased during drying. (4) Compounds such as 2-ethyl-1,4-dimethylbenzene, 1-cthyl-2-methylbenzene, *(3E)*-non-3-en-1-ol, *(3E)*-8-methylnona-3,7-dien-2-one, 4-methylhexyl 3-methylbutanoate, 3,5,5-trimethylhexan-1-ol, 1,2,4,5-tetramethylbenzene, and heptyl butanoate that decreased significantly after steaming, remained stable after rolling and shaping and increased significantly after drying. (5) Compounds such as heptan-2-one, dihydro-β-ionone, *cis*-3-hexenyl hexanoate, 2-butyloct-2-enal, 2-phenylacetaldehyde, linalool, and phenylmethanol that decreased significantly after steaming and rolling processes but increased significantly during shaping and drying processes.

The aroma quality of tea is significantly influenced by many components. *(2E)*-non-2-enal, an important aroma component in Longjing tea with a pleasant fragrance at low concentrations but unpleasant odor at high concentrations [26], is a major constituent in relatively higher proportions in Enshi Yulu tea (Table 2). Acetophenone, a volatile metabolite of phenylalanine [42], is a compound with higher relative content in Laoshan green and Longjing teas, with a pleasant, sweet, and floral aroma [24,25]. 6-methyl-5-hepten-2-one, derived from the degradation of carotenoids, is a compound with higher relative content in Longjing green tea, mainly with a floral fragrance [23]. Both oct-1-en-3-ol and *cis*-3-hexenyl hexanoate are derived from fatty acid degradation [4]; the former smells of mushrooms, and the latter has a fresh and fruity aroma, and both are important aroma components of Longjing green tea [25]. Enshi Yulu tea also contains a relatively high proportion of these ingredients (Table 2). Phenylmethanol and 2-phenylacetaldehyde are volatile products derived from the metabolism of phenylalanine [43]. Phenylmethanol and 2-phenylacetaldehyde mainly have floral and fruity aromas, which are compounds with higher relative contents in green tea [23,25]. Our study found that they also have relatively high content in Enshi Yulu tea (Table 2). Longifolene is mainly sweet and rose and is an important aroma component in chestnut-green tea [13]. However, it has been reported that the concentration of longifolene significantly increases following the dehydration process of green tea [13], contrary to our results. Ethylbenzene is also a compound with a higher relative content of chestnut-green tea [27]. Additionally, safranal mainly presents a herbal and woody aroma, which is an important aroma component in Pu’er tea [44] and Yunnan black teas [28]. Dihydro-β-ionone has a delightful floral fragrance and is a significant aromatic constituent in Pu-erh ripe tea [45]; 4-methyl-3-penten-2-one has a pleasant and sweet aroma and is a significant aromatic component of black tea [46]; and *(2E,4E)*-octa-2,4-dienal plays a significant role as an aromatic constituent in Yunnan black tea [31]. Based on the relative contents of these aroma components, odor characteristics, and previous research, we infer that alterations in phenylmethanol, 2-phenylacetaldehyde, *(2E)*-non-2-enal, oct-1-en-3-ol, and *cis*-3-hexenyl hexanoate exert the most significant impact on the aroma profile of Enshi Yulu tea during processing.

## 4. Conclusions

We used HS-SPME-GC/MS to analyze the aroma components of Enshi Yulu tea during processing. The 242 identified volatile compounds included 45 esters, 43 alcohols, 41 ketones, 32 alkenes, 20 aldehydes, 18 alkanes, 15 aromatic hydrocarbons, 10 oxygen heterocycles, 7 lactones, 6 nitrogen heterocycles, and 5 acids. From fresh leaves to the shaping stage, there were significant decreases in the contents of overall aroma substances, although these increased after drying. Linalool had the highest relative content (12.35%), followed by compounds such as geraniol (7.41%), 2,6-dimethylhept-5-enal (6.93%), phenylmethanol (5.98%), isobutyl acetate (4.16%), hexan-1-ol (3.95%), 2-phenylacetaldehyde (3.80%), and oct-1-en-3-ol (3.33%). During the steaming process, there were 159 differential volatile components, of which 20 were up-regulated, and 139 were down-regulated. During the rolling process, all 24 differential volatile components were down-regulated; of 116 differential volatile components during the shaping process, 60 were up-regulated, and 51 were down-regulated; during the drying process, 68 of the 81 differential volatile components were up-regulated, and 13 were down-regulated. Most variation occurred after steaming, and the least variation occurred after rolling.

PLS-DA analysis revealed significant variation in aroma components during the processing stages of Enshi Yulu tea and identified 100 compounds with higher relative contents. These compounds followed five distinct patterns of change, with phenylmethanol, 2-phenylacetaldehyde, *(2E)*-non-2-enal, oct-1-en-3-ol, and *cis*-3-hexenyl hexanoate exerting a profound impact on the overall aroma quality. These findings improve our knowledge of chemical theories related to tea aroma quality and deepen our understanding of mechanisms responsible for aroma formation in steamed green teas.

## Figures and Tables

**Table 1:** The proportion of different classes of volatiles in the processing stage of Enshi Yulu tea.

Class	FTL	STL	RTL	SPTL	DTL
Aromatics	1.35 ± 0.01 c	1.46 ± 0.09 b	1.40 ± 0.02 b	1.93 ± 0.02 a	1.40 ± 0.01 b
Alkane	2.48 ± 0.03 d	3.09 ± 0.20 c	3.30 ± 0.04 b	3.01 ± 0.05 c	4.79 ± 0.08 a
Alkene	7.35 ± 0.04 b	6.33 ± 0.40 c	6.73 ± 0.10 c	6.44 ± 0.12 c	7.97 ± 0.06 a
Alcohol	43.11 ± 0.15 a	37.61 ± 2.40 b	38.25 ± 0.33 b	39.32 ± 0.32 b	39.68 ± 0.16 b
Aldehyde	14.23 ± 0.14 d	14.98 ± 0.97 cd	17.43 ± 0.11 a	16.86 ± 0.37 b	15.08 ± 0.09 c
Ketone	9.24 ± 0.16 d	9.98 ± 0.60 c	11.50 ± 0.19 b	12.36 ± 0.40 a	12.25 ± 0.25 a
Acid	0.79 ± 0.03 a	0.45 ± 0.04 c	0.60 ± 0.07 b	0.27 ± 0.02 e	0.34 ± 0.02 d
Ester	11.94 ± 0.12 a	9.94 ± 0.62 c	10.46 ± 0.04 b	9.69 ± 0.13 c	9.19 ± 0.06 d
Lactone	6.67 ± 0.07 b	7.66 ± 0.45 a	7.60 ± 0.10 a	5.58 ± 0.03 c	4.47 ± 0.12 d
Oxygen heterocycle	1.34 ± 0.04 c	0.85 ± 0.06 e	0.98 ± 0.05 d	1.68 ± 0.02 b	1.99 ± 0.03 a
Nitrogen heterocycle	1.51 ± 0.02 c	1.78 ± 0.12 b	1.76 ± 0.02 b	2.84 ± 0.02 a	2.84 ± 0.07 a

Note: All values are shown as mean ± SD. Distinct letters (a–e) within the identical row signify significant variations among the processing stage of Enshi Yulu tea (*p* < 0.05). FTL, fresh tea leaves; STL, steamed tea leaves; RTL, rolled tea leaves; SPTL, shaped tea leaves; DTL, dried tea leaves.

**Table 2:** Top 40 volatiles (proportion ≥ 0.44%) in Enshi Yulu tea (DTL) and their odors.

Compounds	Proportion (%)	Odor Characteristic	Compounds	Proportion (%)	Odor Characteristic
linalool	12.35	floral ^a^	*(3Z)*-hex-3-en-1-ol	1.01	fragrant, grassy ^a^
geraniol	7.41	floral, sweet ^a^	2,3-dimethylhexane	0.94	
2,6-dimethylhept-5-enal	6.93		3,3,6-trimethylhepta-1,5-dien-4-one	0.94	
phenylmethanol	5.98	floral, sweet ^a^	tetradecane	0.93	
isobutyl acetate	4.16		3-methylcumarin	0.91	
hexan-1-ol	3.95	fragrant, grassy ^a^	*cis*-3-hexenyl valerate	0.82	fruity, apple ^f^
2-phenylacetaldehyde	3.80	floral, fatty ^b^	*trans*-linalool oxide (pyranoid)	0.78	floral ^g^
oct-1-en-3-ol	3.34	sewant mushrooms ^b^	benzaldehyde	0.71	fruity, fragrant, mint ^b^
*p*-menthone	2.99	mint ^c^	*cis*-3-hexenyl hexanoate	0.69	fruity, fragrant ^b^
β-ionone	2.21	floral, woody ^a^	2-methylisoborneol	0.65	
*(2E)*-non-2-enal	2.19	fragrant, stinkbug ^b^	2,3,3-trimethyloctane	0.63	
dehydromevalonic lactone	2.09		naphthalene	0.62	reagent, kerosene ^b^
abhexone	1.86	spice, spicy ^d^	*(Z)*-3-hexenyl propanoate	0.61	
β-ionone-5,6-epoxide	1.83	fruity ^e^	decane	0.55	
*(5E)*-2,6-dimethylocta-5,7-dien-2-ol	1.77		*trans*-calamenene	0.54	fragrant, grassy ^a^
2,2,3-trimethylcyclobutan-1-one	1.66		hexanal	0.53	
2-ethoxy-3-methylpyrazine	1.23		5-ethyl-2(5H)-furanone	0.51	
*trans*-linalool oxide (furanoid)	1.20	floral, sweet ^a^	hexyl 3-methylbutanoate	0.49	fruity ^h^
methylethylmaleimide	1.07		α-terpinolene	0.47	
2-methylundecane	1.03		lilac aldehyde C	0.44	

Note: a (Ho et al., 2015) [20]; b (Wang et al., 2020) [26]; c (Iwasa et al., 2015) [31]; d (Baniţă et al., 2023) [32]; e (Rigling et al., 2022) [33]; f (Ma et al., 2022) [28]; g (Xu et al., 2016) [34]; h (Heng et al., 2023) [35].

## Data Availability

The original contributions presented in the study are included in the article/Supplementary material, further inquiries can be directed to the corresponding author.

## Supplementary Materials

The following supporting information can be downloaded at https://www.mdpi.com/article/10.3390/foods13233968/s1. Table S1: List of 242 identified volatile compounds and their contents during the processing of Enshi Yulu tea; Table S2: The differential votalitle compound in the processing stages of Enshi Yulu tea; Figure S1. CV distribution of each group; Figure S2. The essential spectrum detection of TIC overlap in the QC sample.
