# Supplementary material for: Analysis of the Changes in Volatile Components During the Processing of Enshi Yulu Tea
Source: Foods. 2024 Dec 9;13(23):3968. doi: 10.3390/foods13233968 (PMC11641255; doi:10.3390/foods13233968)
Supplement: Supplementary file 1 [file foods-13-03968-s001.zip › Supplementary File-Table S1.pdf]

Table S1. List of 242 identified volatile compounds and their contents during the processing of Enshi Yulu tea

NO.	CAS	Compounds	Class	NIST_RI	FTL (ug/kg)	STL (ug/kg)	RTL (ug/kg)	SPTL (ug/kg)	DTL (ug/kg)	VIP value
1	100-41-4	ethylbenzene	Aromatics	855	31.69±0.89	44.91±1.45	53.77±0.70	94.06±3.10	49.20±0.97	1.30
2	95-47-6	1,2-xylene	Aromatics	888	27.56±1.28	30.08±1.86	29.96±0.77	38.91±1.32	34.27±0.84	1.18
3	100-42-5	ethenylbenzene	Aromatics	893	56.05±3.46	83.00±5.37	108.34±2.45	203.88±4.98	107.43±1.31	1.29
4	611-14-3	1-ethyl-2-methylbenzene	Aromatics	971	5.74±0.20	4.01±0.15	3.49±0.14	4.07±0.26	7.13±0.25	1.03
5	98-83-9	(prop-1-en-2-yl)benzene	Aromatics	986	5.68±0.34	6.01±0.35	5.93±0.26	4.66±0.51	7.00±0.15	1.11
6	1758-88-9	2-ethyl-1,4-dimethylbenzene	Aromatics	1076	25.54±1.25	18.85±0.24	17.12±0.29	16.48±0.44	23.48±0.54	1.02
7	95-93-2	1,2,4,5-tetramethylbenzene	Aromatics	1115	16.01±0.62	12.13±0.27	10.83±0.16	11.63±0.39	22.90±0.45	1.11
8	637-69-4	1-ethenyl-4-methoxybenzene	Aromatics	1156	17.07±0.43	9.43±0.36	8.06±0.24	7.84±0.20	12.53±0.31	0.85
9	91-20-3	naphthalene	Aromatics	1182	496.17±17.84	458.21±20.89	266.81±6.75	276.51±11.05	243.15±11.65	1.43
10	54410-74-1	1-methyl-2-(pentan-3-yl)benzene	Aromatics	1240	74.12±2.79	34.06±1.57	25.31±0.69	16.39±0.39	13.08±0.82	0.72
11	91-57-6	2-methylnaphthalene	Aromatics	1297	53.73±1.49	33.38±3.16	20.02±0.40	27.96±1.64	24.70±2.08	0.97
12	575-43-9	1,6-dimethylnaphthalene	Aromatics	1422	25.22±0.67	5.72±0.62	3.33±0.28	3.61±0.48	3.29±0.46	0.71
13	643-93-6	3-methyl-1,1'-biphenyl	Aromatics	1485	4.97±0.39	2.49±0.17	2.81±0.27	1.18±0.15	1.64±0.14	0.81
14	54932-91-1	(4-methyloctan-4-yl)benzene	Aromatics	1504	42.21±1.67	16.50±1.00	14.77±0.18	20.43±1.31	16.76±1.42	0.77
15	483-78-3	1,6-dimethyl-4-isopropylnaphthaIene	Aromatics	1674	10.46±0.17	5.25±0.37	4.90±0.25	7.63±0.43	6.53±0.43	0.93
16	584-94-1	2,3-dimethylhexane	Alkane	760	18.28±1.06	302.84±13.43	210.34±5.30	324.69±13.20	367.83±13.47	1.03
17	111-84-2	nonane	Alkane	900	30.86±1.33	27.48±1.20	20.59±0.50	41.95±1.01	33.09±0.44	1.49
18	871-83-0	2-methylnonane	Alkane	964	4.98±0.54	7.97±0.35	6.06±0.09	5.29±0.44	4.26±0.45	1.32
19	62016-30-2	2,3,3-trimethyloctane	Alkane	966	357.31±15.64	358.07±6.66	352.92±4.15	260.75±8.22	243.84±4.62	1.05
20	33626-25-4	trans-pinane	Alkane	972	103.38±4.15	75.03±3.85	54.65±1.09	35.56±0.78	28.89±1.56	0.88
21	124-18-5	decane	Alkane	1000	53.05±3.75	66.99±1.78	53.73±1.00	58.76±2.78	213.27±5.11	1.10
22	17302-28-2	2,6-dimethylnonane	Alkane	1018	12.03±1.40	16.60±0.33	14.22±0.20	11.44±1.03	10.16±0.36	1.11
23	2847-72-5	4-methyldecane	Alkane	1060	141.14±7.11	112.82±2.30	100.46±1.37	77.65±1.97	69.65±2.60	0.85
24	62108-22-9	2,5,9-trimethyldecane	Alkane	1121	4.69±0.42	5.44±0.18	5.53±0.24	7.52±0.32	12.52±0.11	0.93
25	7045-71-8	2-methylundecane	Alkane	1164	642.36±70.11	440.67±25.80	363.49±24.20	169.63±5.40	401.68±11.78	1.05
26	17312-81-1	3,5-dimethylundecane	Alkane	1207	46.69±5.47	41.45±1.50	44.78±1.87	34.35±1.63	86.33±2.30	1.15
27	629-50-5	tridecane	Alkane	1300	17.83±1.55	7.62±0.46	8.63±0.30	5.35±0.31	14.92±0.64	0.98
28	629-59-4	tetradecane	Alkane	1400	98.57±9.53	42.18±2.00	38.10±1.16	25.01±1.00	361.62±25.68	1.12
29	62238-11-3	2,3,5-trimethyldecane	Alkane	1411	20.91±1.54	9.93±0.62	9.49±0.32	5.79±0.25	8.57±0.38	0.73
30	3891-99-4	2,6,10-trimethyltridecane	Alkane	1449	11.85±1.10	4.06±0.17	4.37±0.30	2.58±0.19	3.42±0.22	0.73
31	25117-24-2	4-methyltetradecane	Alkane	1459	52.94±3.71	81.33±4.95	67.82±3.35	77.47±6.45	73.80±4.15	1.17
32	629-62-9	pentadecane	Alkane	1500	14.64±1.26	5.10±0.40	6.17±0.53	3.31±0.09	7.13±0.37	0.83
33	1560-93-6	2-methylpentadecane	Alkane	1563	6.12±0.50	1.93±0.18	2.05±0.22	0.98±0.09	17.22±1.49	1.11
34	111-66-0	oct-1-ene	Alkene	789	111.15±4.94	17.44±1.60	14.04±1.51	15.53±0.84	66.05±2.92	0.85
35	80-56-8	α -pinene	Alkene	937	8.50±0.64	8.05±1.14	7.04±0.20	10.85±0.98	6.94±0.35	1.32
36	6671-66-5	bicyclo[3.3.1]non-2-ene	Alkene	964	113.24±5.36	112.15±1.41	112.82±1.23	86.67±2.03	85.23±1.09	1.11
37	99-86-5	α -terpinene	Alkene	1012	21.49±1.49	13.50±0.90	8.84±0.43	7.85±0.80	8.35±0.63	0.85

NO.	CAS	Compounds	Class	NIST_RI	FTL (ug/kg)	STL (ug/kg)	RTL (ug/kg)	SPTL (ug/kg)	DTL (ug/kg)	VIP value
38	62108-28-5	4,8-dimethylnona-1,7-diene	Alkene	1026	43.38±1.69	3.91±0.24	3.22±0.18	3.59±0.23	15.49±0.68	0.78
39	586-62-9	α -terpinolene	Alkene	1073	318.95±18.35	160.41±6.26	89.84±1.60	83.03±3.05	183.99±7.16	0.93
40	19945-61-0	(3E)-4,8-dimethylnona-1,3,7-triene	Alkene	1116	14.58±0.59	20.13±1.14	14.10±0.67	18.58±1.58	35.89±0.91	1.19
41	74630-41-4	9-methylundec-1-ene	Alkene	1152	3.33±0.59	127.78±4.06	111.82±2.27	94.48±1.34	109.66±6.10	1.00
42	112-41-4	dodec-1-ene	Alkene	1190	893.70±41.30	551.15±33.33	381.66±7.06	197.53±8.99	127.66±9.45	0.82
43	17699-14-8	α -cubebene	Alkene	1351	29.42±1.03	10.11±0.61	10.39±0.24	18.55±1.28	17.97±1.38	0.93
44	1137-12-8	longicyclene	Alkene	1374	9.56±0.41	2.17±0.17	2.59±0.12	4.48±0.22	3.93±0.42	0.88
45	3856-25-5	copaene	Alkene	1376	51.15±1.01	29.65±1.39	28.25±0.66	48.37±2.86	44.05±2.99	1.10
46	5208-59-3	β -bourbonene	Alkene	1384	11.23±0.82	7.00±0.62	7.55±0.55	15.39±1.58	18.40±1.55	1.04
47	13744-15-5	β -cubebene	Alkene	1390	27.43±1.09	9.43±0.75	7.25±0.21	11.30±0.98	10.65±0.70	0.75
48	94482-89-0	isoitalicene	Alkene	1395	9.75±0.39	1.70±0.26	1.89±0.14	3.28±0.38	3.13±0.32	0.81
49	475-20-7	longifolene	Alkene	1406	9.69±0.15	4.79±0.31	5.10±0.14	9.32±0.51	6.88±0.55	1.21
50	489-40-7	α -gurgujene	Alkene	1409	10.29±0.19	3.81±0.20	4.38±0.12	8.37±0.60	6.60±0.57	1.11
51	546-28-1	β -cedrene	Alkene	1421	28.57±0.51	31.53±1.62	15.02±0.41	10.64±0.67	5.73±0.38	1.45
52	6831-16-9	aristolene	Alkene	1447	19.79±1.00	5.96±0.35	8.35±0.92	6.74±1.06	7.07±0.70	0.87
53	5090-61-9	nootkatene	Alkene	1456	8.33±0.50	2.08±0.15	2.12±0.07	3.45±0.33	3.04±0.19	0.80
54	18794-84-8	(E)-β-famesene	Alkene	1457	7.91±0.77	11.12±0.62	9.29±0.47	11.40±0.85	11.15±0.77	1.08
55	157477-72-0	cis-4(14),5-muuroladiene	Alkene	1463	24.40±1.36	5.27±0.40	6.12±0.31	8.44±0.80	7.02±0.57	0.80
56	26560-14-5	(Z, E)-α-farnesene	Alkene	1491	557.14±18.84	218.28±23.28	229.96±35.68	58.55±8.86	84.09±10.90	0.76
57	10208-80-7	α -Muurolene	Alkene	1499	14.03±0.77	3.11±0.37	3.41±0.18	5.53±0.50	4.72±0.32	0.82
58	78204-62-3	dehydro-ar-α-himachalene	Alkene	1516	46.10±1.30	24.38±1.77	25.31±0.74	45.02±3.26	33.56±2.60	1.22
59	483-76-1	δ -cadinene	Alkene	1524	312.03±9.06	124.01±9.84	109.62±2.84	173.78±13.62	133.67±11.68	0.83
60	20307-83-9	β -sesquiphellanderene	Alkene	1524	13.46±0.57	5.93±0.35	5.13±0.27	7.54±0.75	7.66±0.44	0.78
61	73209-42-4	trans-calamenene	Alkene	1529	377.90±12.92	136.04±9.83	145.61±4.02	272.53±19.83	208.44±17.19	1.04
62	29837-12-5	cubenene	Alkene	1532	48.55±1.31	13.01±1.13	14.86±0.41	24.71±2.33	15.99±1.25	0.90
63	24406-05-1	α -cadinene	Alkene	1538	32.35±1.15	8.77±0.69	10.05±0.30	16.85±1.57	11.31±0.98	0.91
64	27840-40-0	β -vetivenene	Alkene	1540	4.08±0.13	1.46±0.16	1.80±0.05	3.61±0.36	2.15±0.25	1.21
65	50277-34-4	β -calacorene	Alkene	1563	35.19±1.03	16.30±1.19	14.27±0.34	20.76±1.32	17.60±1.69	0.81
66	71-41-0	pentan-1-ol	Alcohol	766	159.03±3.36	154.83±3.05	157.17±1.18	158.73±4.62	159.58±8.75	0.60
67	111-27-3	hexan-1-ol	Alcohol	780	1617.41±13.53	1576.94±7.21	1494.83±4.63	1550.52±18.26	1540.60±19.95	1.35
68	928-99-1	(3Z)-hex-3-en-1-ol	Alcohol	868	716.95±15.09	387.46±42.02	337.67±21.89	205.62±27.49	394.55±9.29	0.81
69	590-67-0	1-methylcyclohexan-1-ol	Alcohol	908	22.72±0.94	19.98±0.88	15.06±0.52	29.66±0.27	24.64±0.63	1.49
70	3391-86-4	oct-1-en-3-ol	Alcohol	982	154.47±5.32	153.76±7.83	110.48±2.36	89.76±4.11	1301.04±62.48	1.12
71	1569-60-4	6-methylhept-5-en-2-ol	Alcohol	994	7.91±0.61	6.82±0.34	5.38±0.36	6.54±0.25	6.05±0.22	1.14
72	123-96-6	octan-2-ol	Alcohol	998	5.34±0.40	5.76±0.27	3.74±0.25	6.02±0.26	4.53±0.09	1.82
73	5441-52-1	3,5-dimethylcyclohexan-1-ol	Alcohol	1030	5.12±0.15	3.19±0.54	2.41±0.22	2.74±0.09	2.27±0.16	0.74
74	104-76-7	2-ethylhexan-1-ol	Alcohol	1030	10.58±0.52	4.92±0.31	3.53±0.16	3.58±0.18	25.77±0.99	1.13
75	100-51-6	phenylmethanol	Alcohol	1036	1316.78±56.48	1140.85±69.74	821.50±20.56	2052.56±56.08	2331.43±107.32	1.11
76	3452-97-9	3,5,5-trimethylhexan-1-ol	Alcohol	1047	12.98±0.69	6.72±0.23	5.73±0.29	5.95±0.23	25.92±1.27	1.10
77	98-85-1	1-phenylethan-1-ol	Alcohol	1061	23.08±1.03	13.00±0.27	12.75±0.18	9.80±0.35	8.70±0.68	0.72

NO.	CAS	Compounds	Class	NIST_RI	FTL (ug/kg)	STL (ug/kg)	RTL (ug/kg)	SPTL (ug/kg)	DTL (ug/kg)	VIP value
78	18409-17-1	(2E)-oct-2-en-1-ol	Alcohol	1067	30.03±2.14	14.34±0.71	9.96±0.22	14.91±0.67	11.12±0.19	0.80
79	34995-77-2	trans-linalool oxide (furanoid)	Alcohol	1086	540.69±32.80	221.07±21.15	197.96±4.70	334.68±8.16	465.63±5.24	0.92
80	767-05-5	3-cyclopentylpropan-1-ol	Alcohol	1102	160.08±10.95	91.67±3.32	67.61±2.54	110.75±2.70	92.95±1.30	0.96
81	628-99-9	nonan-2-ol	Alcohol	1102	35.15±1.36	26.57±0.89	19.24±1.19	34.86±0.93	29.05±0.58	1.41
82	35192-73-5	non-1-en-4-ol	Alcohol	1103	230.49±11.27	122.58±4.37	90.89±3.12	160.64±4.06	132.55±1.72	1.00
83	78-70-6	linalool	Alcohol	1106	3886.56±131.55	3782.49±123.27	3524.22±82.29	4720.75±110.90	4813.25±63.41	1.08
84	10339-61-4	(3E)-non-3-en-1-ol	Alcohol	1143	7.29±0.65	2.80±0.31	2.12±0.18	2.41±0.38	11.95±0.19	1.10
85	696-71-9	cyclooctanol	Alcohol	1155	9.23±1.16	28.87±0.58	27.55±0.48	23.38±0.45	35.27±0.67	0.95
86	56805-23-3	(3Z,6Z)-nona-3,6-dien-1-ol	Alcohol	1156	5.38±0.58	93.90±2.81	79.08±1.01	59.60±0.79	49.32±3.30	1.07
87	31502-19-9	(6E)-non-6-en-1-ol	Alcohol	1167	330.85±33.76	212.55±9.84	179.83±10.05	87.93±2.93	169.30±4.68	0.92
88	7643-60-9	(5E)-2,6-dimethylocta-5,7-dien-2-ol	Alcohol	1169	71.64±8.81	1234.75±37.31	1054.84±14.28	787.67±12.32	688.88±37.06	1.07
89	498-16-8	5-methyl-2-(prop-1-en-2-yl)hex-4-en-1-ol	Alcohol	1170	152.50±7.71	91.57±4.99	72.88±1.61	60.48±0.65	58.89±1.68	0.75
90	39028-58-5	trans-linalool oxide (pyranoid)	Alcohol	1173	837.73±40.68	474.35±25.08	343.87±5.80	304.04±5.06	302.42±9.75	0.76
91	143-08-8	nonan-1-ol	Alcohol	1173	69.31±3.97	37.52±2.28	32.13±0.68	24.29±0.64	27.15±0.82	0.74
92	562-74-3	terpinen-4-ol	Alcohol	1177	261.64±19.60	32.73±1.21	35.88±1.10	24.45±0.55	50.03±1.18	0.73
93	27779-29-9	isopinocampheol	Alcohol	1179	19.09±0.34	13.54±0.77	10.78±0.26	8.73±0.40	104.88±1.77	1.12
94	10482-56-1	α -terpineol	Alcohol	1190	67.10±2.36	31.11±4.53	21.57±2.65	20.07±0.85	35.67±3.11	0.79
95	515-00-4	myrtenol	Alcohol	1195	125.32±5.31	51.97±2.80	39.46±1.38	20.47±1.44	16.98±1.50	0.71
96	2371-42-8	2-methylisoborneol	Alcohol	1198	1348.72±53.48	823.86±44.66	599.47±11.08	332.62±12.34	251.67±18.73	0.80
97	74410-00-7	trans-isopiperitenol	Alcohol	1210	4.04±0.50	3.62±0.12	3.84±0.21	2.71±0.11	11.33±0.26	1.15
98	99-48-9	carveol	Alcohol	1219	2.67±0.18	3.56±0.26	1.73±0.11	1.97±0.16	4.65±0.41	1.61
99	1117-61-9	β -citronellol	Alcohol	1220	58.25±1.91	9.63±0.78	5.28±0.20	4.92±0.24	8.41±0.42	0.72
100	106-24-1	geraniol	Alcohol	1229	15918.21±669.68	8589.09±488.59	6249.75±130.37	3620.59±80.91	2888.56±187.74	0.76
101	112-30-1	decan-1-ol	Alcohol	1272	29.24±3.25	16.14±0.59	18.93±0.87	12.44±0.76	21.75±0.64	0.98
102	490-03-9	diosphenol	Alcohol	1302	1.93±0.09	1.13±0.10	0.72±0.07	0.78±0.07	5.12±0.16	1.14
103	1946-00-5	limonene-1,2-diol	Alcohol	1321	60.29±5.50	29.98±0.99	33.37±1.53	20.50±0.66	30.56±0.75	0.83
104	103619-06-3	cis-8-hydroxylinalool	Alcohol	1361	11.64±0.67	5.46±0.27	5.37±0.20	3.79±0.21	39.08±2.80	1.12
105	13019-16-4	2-butyloct-2-enal	Alcohol	1378	15.59±1.10	11.92±0.52	13.47±0.62	26.55±1.82	26.25±2.30	1.12
106	40642-40-8	(8Z)-dodec-8-en-1-ol	Alcohol	1468	9.09±0.53	13.95±0.75	11.75±0.50	14.64±1.23	13.41±0.68	1.06
107	69064-37-5	(2E)-dodec-2-en-1-ol	Alcohol	1478	122.47±5.45	47.21±4.55	51.59±6.91	16.76±2.00	12.94±2.05	0.76
108	73365-77-2	di-epi-1,10-cubenol	Alcohol	1614	15.51±0.81	7.56±0.49	3.30±0.14	3.31±0.29	2.73±0.24	0.87
109	66-25-1	hexanal	Aldehyde	801	444.47±26.63	214.46±13.83	261.39±11.61	228.62±17.62	206.41±7.93	0.92
110	17844-21-2	4-methylidenehex-5-enal	Aldehyde	897	10.77±0.71	9.40±0.30	6.41±0.13	13.50±0.74	12.06±0.22	1.44
111	62238-34-0	(4E)-hept-4-enal	Aldehyde	899	15.51±0.88	11.26±1.12	13.10±0.39	26.36±1.22	21.78±0.38	1.25
112	111-71-7	heptanal	Aldehyde	903	80.82±3.87	74.09±2.89	51.93±1.30	105.84±2.44	86.32±1.22	1.57
113	100-52-7	benzaldehyde	Aldehyde	960	354.51±21.00	121.39±3.32	117.69±1.26	163.87±4.91	274.74±6.48	0.86
114	122-78-1	2-phenylacetaldehyde	Aldehyde	1049	1245.64±37.73	793.94±35.10	950.39±33.10	1418.16±82.55	1479.83±21.05	1.16
115	106-72-9	2,6-dimethylhept-5-enal	Aldehyde	1054	5828.35±284.23	4413.36±93.40	3975.90±46.15	3002.27±50.21	2701.83±110.46	0.82
116	2548-87-0	(2E)-oct-2-enal	Aldehyde	1060	39.35±1.55	35.58±0.76	33.03±0.37	33.78±0.87	26.99±1.03	1.02
117	35158-25-9	5-methyl-2-(propan-2-yl)hex-2-enal	Aldehyde	1106	198.99±6.70	117.37±3.65	90.58±2.76	149.99±3.51	127.39±1.22	1.05

NO.	CAS	Compounds	Class	NIST_RI	FTL (ug/kg)	STL (ug/kg)	RTL (ug/kg)	SPTL (ug/kg)	DTL (ug/kg)	VIP value
118	30361-28-5	(2E,4E)-octa-2,4-dienal	Aldehyde	1115	3.25±0.05	3.51±0.29	2.51±0.25	3.84±0.38	5.50±0.32	1.13
119	432-24-6	α -cyclocitral	Aldehyde	1116	2.83±0.20	1.89±0.15	2.00±0.18	1.43±0.33	2.85±0.15	0.94
120	18829-56-6	(2E)-non-2-enal	Aldehyde	1162	65.75±9.53	1361.24±42.29	1178.37±20.33	993.93±21.85	853.68±44.41	1.01
121	53447-48-6	lilac aldehyde C	Aldehyde	1167	470.39±47.56	274.77±13.37	230.40±14.45	105.09±3.04	172.14±7.13	0.82
122	27841-22-1	3-p-menthen-7-al	Aldehyde	1169	35.59±1.62	26.03±1.19	22.49±0.63	22.35±0.26	24.02±0.95	0.80
123	15764-16-6	2,4-dimethylbenzaldehyde	Aldehyde	1182	2.34±0.15	2.44±0.09	2.05±0.14	2.65±0.10	3.18±0.16	1.07
124	116-26-7	safranal	Aldehyde	1201	3.37±0.32	3.94±0.26	3.21±0.15	3.49±0.27	9.49±0.48	1.14
125	21661-97-2	(7Z)-dec-7-enal	Aldehyde	1212	3.95±0.42	5.98±0.29	5.70±0.29	4.47±0.17	14.05±0.53	1.13
126	106-26-3	neral	Aldehyde	1240	587.46±23.79	295.93±16.86	220.10±4.23	131.19±2.47	111.72±7.12	0.74
127	141-27-5	geranial	Aldehyde	1273	15.11±2.67	15.00±0.47	17.87±0.71	12.04±0.66	22.82±1.06	1.15
128	112-45-8	undec-10-enal	Aldehyde	1297	4.40±0.31	3.17±0.17	2.64±0.09	2.76±0.08	11.92±0.31	1.12
129	6704-19-4	1-cyclopropylpropan-1-one	Ketone	756	26.27±1.52	44.30±1.77	30.74±0.62	45.30±1.86	52.65±1.68	1.38
130	141-79-7	4-methylpent-3-en-2-one	Ketone	798	30.35±1.76	7.47±0.22	4.74±0.17	5.15±0.36	160.38±6.22	1.11
131	1449-49-6	2,2,3-trimethylcyclobutan-1-one	Ketone	847	681.48±59.48	712.45±35.79	781.80±19.16	701.24±100.97	647.86±41.68	0.55
132	108-94-1	cyclohexanone	Ketone	894	37.68±2.00	32.02±1.00	23.25±0.79	51.27±1.34	41.43±0.79	1.46
133	110-43-0	heptan-2-one	Ketone	895	4.72±0.23	4.23±0.30	2.89±0.08	5.64±0.30	6.56±0.24	1.24
134	3214-41-3	octane-2,5-dione	Ketone	984	14.56±0.49	16.91±0.74	11.94±0.27	11.21±0.37	121.38±5.38	1.11
135	110-93-0	6-methylhept-5-en-2-one	Ketone	986	20.29±0.73	35.25±1.90	23.71±0.47	40.68±0.69	50.74±2.64	1.20
136	54549-81-4	α -thujamenthone	Ketone	1028	48.96±1.69	5.43±0.17	5.19±0.28	5.29±0.18	10.27±0.32	0.75
137	18402-82-9	(3E)-oct-3-en-2-one	Ketone	1035	367.43±13.64	125.10±6.14	82.67±1.24	42.51±1.05	31.53±0.89	0.71
138	2408-37-9	2,2,6-trimethylcyclohexan-1-one	Ketone	1036	73.47±3.98	30.85±1.54	25.45±1.15	21.51±0.59	43.36±1.25	0.79
139	471-01-2	3,5,5-trimethylcyclohex-3-en-1-one	Ketone	1044	28.78±0.99	19.22±0.61	17.86±0.99	13.18±0.51	12.03±0.46	0.72
140	82456-34-6	non-3-en-5-one	Ketone	1052	13.52±0.32	6.55±0.20	5.24±0.22	3.99±0.35	4.99±0.22	0.72
141	546-49-6	3,3,6-trimethylhepta-1,5-dien-4-one	Ketone	1062	803.24±39.58	605.43±13.41	547.83±6.03	415.93±7.18	366.21±14.62	0.82
142	98-86-2	acetophenone	Ketone	1065	17.12±0.76	22.51±0.47	20.93±0.39	26.53±0.81	30.16±0.88	0.89
143	30086-02-3	(3E,5E)-octa-3,5-dien-2-one	Ketone	1073	260.72±10.93	77.30±5.55	41.59±1.76	25.96±1.46	19.84±1.58	0.72
144	546-80-5	thujone	Ketone	1103	834.11±33.64	210.72±5.79	183.28±4.46	118.55±4.00	112.05±1.78	0.71
145	35900-26-6	4-methylnonan-5-one	Ketone	1116	12.17±0.85	11.83±0.20	13.27±0.59	16.38±0.75	59.28±2.05	1.05
146	74630-80-1	4,6-dimethylnona-2,7-dien-5-one	Ketone	1138	2.10±0.26	1.28±0.21	0.74±0.13	1.35±0.17	9.19±0.35	1.09
147	56606-79-2	dec-1-en-3-one	Ketone	1141	1.62±0.11	1.78±0.08	1.46±0.08	1.69±0.10	15.69±0.62	1.09
148	35408-14-1	(3E)-8-methylnona-3,7-dien-2-one	Ketone	1144	11.31±0.35	4.07±0.79	2.84±0.10	2.97±0.17	13.76±0.43	1.05
149	89-80-5	p-menthone	Ketone	1154	100.43±13.05	1978.64±64.30	1712.62±26.83	1420.85±20.99	1164.26±62.18	1.02
150	30460-92-5	pinocarvone	Ketone	1164	32.19±4.15	88.13±2.55	96.16±2.05	90.82±3.73	91.00±5.21	0.86
151	24545-81-1	3-thujen-2-one	Ketone	1171	4.45±0.19	11.09±0.96	13.12±0.75	15.76±1.34	14.72±1.08	0.75
152	577-16-2	1-(2-methylphenyl)ethan-1-one	Ketone	1173	15.50±0.32	11.86±0.31	10.09±0.23	10.26±0.12	13.11±0.49	0.96
153	15358-88-0	isocamphopinone	Ketone	1173	119.43±6.56	68.94±3.54	53.57±1.24	45.87±0.83	45.51±1.38	0.75
154	529-00-0	p-ment-8-en-3-one	Ketone	1179	32.10±2.52	5.53±0.30	5.53±0.17	5.02±0.38	7.40±0.27	0.73
155	3792-53-8	cis-dihydrocarvone	Ketone	1195	512.72±20.23	297.42±15.25	215.49±4.27	116.49±4.88	97.27±5.71	0.79
156	10136-65-9	2-hydroxy-3-pinanone	Ketone	1256	105.87±5.19	51.20±1.45	47.52±1.61	32.51±1.24	59.25±2.31	0.80
157	937-30-4	1-(4-ethylphenyl)ethan-1-one	Ketone	1277	14.45±0.33	6.76±0.39	5.76±0.09	7.09±0.15	7.12±0.55	0.73

NO.	CAS	Compounds	Class	NIST_RI	FTL (ug/kg)	STL (ug/kg)	RTL (ug/kg)	SPTL (ug/kg)	DTL (ug/kg)	VIP value
158	34687-46-2	2-(2-hexenyl)-cyclopentanone	Ketone	1320	21.51±0.90	10.19±0.33	9.55±0.24	5.34±0.20	7.38±0.31	0.75
159	6617-34-1	3-carene-2,5-dione	Ketone	1324	11.39±1.66	4.98±0.34	4.36±0.43	3.86±0.35	3.39±0.29	0.65
160	54868-48-3	(E)-solanone	Ketone	1373	51.24±3.33	10.71±0.42	10.44±0.32	17.96±0.98	16.33±1.20	0.79
161	16183-46-3	2-hydroxy-1-phenylbutan-1-one	Ketone	1390	16.16±0.23	4.07±0.36	3.63±0.17	4.81±0.37	5.29±0.42	0.75
162	488-10-8	cis-jasmone	Ketone	1395	377.48±12.20	30.23±4.50	17.82±0.25	14.78±1.44	14.30±1.28	0.73
163	127-41-3	α -ionone	Ketone	1422	34.09±0.99	9.37±0.93	6.46±0.34	9.76±0.43	11.26±0.56	0.74
164	20483-36-7	7,8-dihydro-3,4-dehydro-β-ionone	Ketone	1424	6.36±0.53	4.59±0.32	3.48±0.23	2.72±0.23	2.73±0.26	0.80
165	29898-25-7	1-phenylhexan-3-one	Ketone	1427	13.45±0.74	8.31±0.84	6.00±0.25	5.39±0.23	5.61±0.35	0.78
166	17283-81-7	dihydro-β-ionone	Ketone	1433	33.18±0.73	9.24±0.82	12.62±0.60	43.40±4.03	58.94±4.12	1.03
167	23267-57-4	β-ionone-5,6-epoxide	Ketone	1473	373.25±10.99	163.65±15.94	174.79±25.68	495.20±67.12	714.53±70.90	0.94
168	79-77-6	β -ionone	Ketone	1491	918.94±25.41	447.28±41.45	503.52±74.72	807.57±54.28	860.00±43.75	0.99
169	79-69-6	6-methyl-α-ionone	Ketone	1526	8.48±0.29	3.37±0.33	3.11±0.13	4.81±0.50	3.70±0.39	0.83
170	79-31-2	2-methylpropanoic acid	Acid	765	10.00±0.22	10.67±0.37	10.37±0.16	12.03±0.37	11.69±0.30	0.87
171	97-61-0	2-methylpentanoic acid	Acid	959	54.17±2.11	37.31±0.67	40.44±2.75	35.58±0.99	42.21±0.81	1.00
172	13419-69-7	(2E)-hex-2-enoic acid	Acid	982	14.74±0.76	9.79±0.40	6.89±0.33	4.03±0.20	3.64±0.25	0.85
173	1123-25-7	1-methylcyclohexane-1-carboxylic acid	Acid	1210	5.25±0.63	7.11±0.39	6.45±0.19	6.00±0.27	19.22±0.72	1.10
174	112-38-9	undecylenic acid	Acid	1484	438.22±15.28	172.12±17.65	181.95±28.04	46.76±7.01	60.27±6.32	0.76
175	110-19-0	isobutyl acetate	Ester	772	1707.05±12.65	1675.85±16.46	1645.27±2.93	1645.63±20.47	1621.60±15.60	0.96
176	110-45-2	3-methylbutyl formate	Ester	792	108.12±4.96	15.30±1.76	12.14±0.92	15.85±0.82	59.82±0.96	0.83
177	141-32-2	butyl acrylate	Ester	861	23.90±6.66	47.12±4.20	48.30±1.92	38.17±5.54	37.88±3.38	0.69
178	1576-85-8	4-pentenyl acetate	Ester	896	17.89±0.87	14.61±0.93	14.77±0.47	28.99±0.73	24.86±0.51	1.21
179	2998-23-4	pentyl prop-2-enoate	Ester	974	95.26±3.82	65.28±3.43	48.24±0.82	24.78±0.67	15.88±0.72	0.85
180	109-21-7	butyl butanoate	Ester	995	30.83±2.42	36.87±1.06	29.97±0.62	31.23±1.72	110.51±2.37	1.11
181	2445-67-2	2-methylpropyl 2-methylbutanoate	Ester	1004	18.00±0.99	18.03±1.02	12.64±0.24	19.74±0.38	13.32±0.35	1.70
182	3681-71-8	(Z)-3-hexenyl acetate	Ester	1005	52.89±4.12	16.88±0.59	13.48±0.51	4.12±0.29	4.70±0.32	0.70
183	2445-69-4	2-methylbutyl isobutyrate	Ester	1016	51.62±6.91	67.78±1.65	58.93±1.11	45.73±3.41	44.47±1.13	1.10
184	2311-46-8	isopropyl hexanoate	Ester	1034	28.07±0.76	8.51±0.27	6.90±0.27	5.04±0.16	12.12±0.32	0.76
185	622-45-7	cyclohexyl acetate	Ester	1043	26.67±0.98	6.93±0.34	7.73±0.53	6.06±0.26	5.92±0.53	0.75
186	27829-72-7	ethyl trans-2-hexenoate	Ester	1049	23.52±1.61	19.28±0.52	18.12±0.54	12.59±0.78	14.69±0.65	0.84
187	106-27-4	3-methylbutyl butanoate	Ester	1056	111.43±12.91	125.73±3.02	115.87±2.58	92.64±5.88	104.90±3.27	0.97
188	10250-45-0	3-methyl-1-isobutylbutyl acetate	Ester	1092	456.85±23.07	309.39±16.31	221.14±4.52	134.80±3.23	104.51±5.48	0.85
189	106-30-9	ethyl heptanoate	Ester	1098	44.11±1.10	17.10±0.77	13.65±0.45	8.10±0.34	7.55±0.21	0.70
190	33467-74-2	(Z)-3-hexenyl propanoate	Ester	1100	269.22±12.56	218.74±6.52	162.42±4.63	283.96±7.68	236.94±1.14	1.49
191	112-06-1	heptyl acetate	Ester	1112	5.34±0.16	2.39±0.26	2.69±0.17	3.55±0.13	14.78±0.72	1.07
192	6290-13-7	cyclopentyl butanoate	Ester	1126	2.68±0.20	3.20±0.09	3.42±0.11	5.68±0.24	10.01±0.35	0.92
193	103-09-3	2-ethylhexyl acetate	Ester	1129	1.21±0.12	1.10±0.12	1.48±0.13	2.41±0.22	3.44±0.17	0.94
194	3289-28-9	cyclopentyl butyrate	Ester	1136	2.45±0.35	1.43±0.10	1.07±0.05	1.55±0.11	11.37±0.56	1.09
195	93-89-0	ethyl benzoate	Ester	1172	121.34±7.39	101.98±2.46	67.69±2.11	51.23±2.01	40.43±1.91	1.08
196	3465-88-1	β -artemisia acetate	Ester	1173	86.47±4.92	47.73±2.00	37.59±2.39	31.29±0.46	28.66±1.77	0.71
197	16491-36-4	cis-3-hexenyl butyrate	Ester	1187	423.79±31.41	52.48±2.56	59.20±1.75	39.86±1.05	47.10±1.13	0.73

NO.	CAS	Compounds	Class	NIST_RI	FTL (ug/kg)	STL (ug/kg)	RTL (ug/kg)	SPTL (ug/kg)	DTL (ug/kg)	VIP value
198	77149-68-9	1-octenyl acetate	Ester	1191	138.68±5.90	82.33±4.96	56.85±1.03	27.10±1.31	18.07±1.46	0.81
199	69668-83-3	(Z)-3-octenyl acetate	Ester	1195	29.76±1.04	17.00±0.59	13.08±0.57	8.43±0.37	8.14±0.56	0.75
200	112-14-1	octyl acetate	Ester	1210	6.75±0.65	6.00±0.30	6.37±0.26	4.91±0.23	13.07±0.31	1.16
201	939-48-0	isopropyl benzoate	Ester	1217	23.88±1.06	9.04±0.81	4.94±0.33	3.12±0.38	2.79±0.30	0.73
202	39026-94-3	1-methylhexyl butanoate	Ester	1218	29.17±2.61	92.92±6.77	45.80±0.52	41.50±1.16	74.47±3.53	1.89
203	35852-46-1	cis-3-hexenyl valerate	Ester	1237	1699.67±71.31	909.00±50.16	667.58±13.45	392.31±8.56	321.28±21.03	0.75
204	10032-13-0	hexyl 3-methylbutanoate	Ester	1244	1160.28±54.52	631.72±35.71	422.95±7.76	241.50±9.69	190.57±11.92	0.78
205	540-07-8	pentyl hexanoate	Ester	1287	6.09±0.58	4.58±0.24	5.02±0.30	3.70±0.29	6.96±0.32	1.09
206	5870-93-9	heptyl butanoate	Ester	1296	60.07±5.28	37.51±0.87	36.28±1.14	29.86±1.10	95.43±4.40	1.12
207	105-86-2	geranyl formate	Ester	1301	18.16±1.50	4.07±0.29	4.74±0.32	2.80±0.19	7.38±0.35	0.83
208	1215127-80-2	4-methylhexyl 3-methylbutanoate	Ester	1312	3.54±0.40	1.17±0.16	0.89±0.11	1.01±0.17	6.10±0.29	1.08
209	2349-14-6	methyl geranate	Ester	1323	39.64±0.59	79.70±7.60	26.12±0.68	5.71±0.30	5.90±0.45	1.75
210	7367-83-1	methyl (4Z)-dec-4-enoate	Ester	1323	23.85±4.48	11.57±0.90	10.63±0.98	9.65±1.27	8.85±0.73	0.58
211	5413-59-2	cyclopentyl hexanoate	Ester	1325	20.87±0.75	10.43±0.49	8.38±0.29	4.66±0.20	6.62±0.29	0.75
212	134-20-3	methyl anthranilate	Ester	1343	23.40±0.80	7.94±0.51	8.55±0.14	15.90±0.97	15.32±1.17	0.98
213	31501-11-8	cis-3-hexenyl hexanoate	Ester	1380	202.61±12.26	120.86±10.92	124.31±4.23	246.85±18.93	267.84±24.53	1.11
214	61444-38-0	cis-3-hexenyl cis-3-hexenoate	Ester	1389	45.39±3.49	15.24±1.38	15.05±1.06	18.88±1.46	12.66±1.06	0.80
215	69727-42-0	2-ethylundecanoate	Ester	1417	22.84±2.16	10.96±0.39	9.60±0.62	6.02±0.37	8.50±0.40	0.72
216	78-36-4	linalyl butyrate	Ester	1418	16.41±0.22	19.02±0.99	9.35±0.24	7.30±0.20	3.84±0.26	1.50
217	2345-24-6	neryl isobutyrate	Ester	1475	535.18±19.60	203.81±21.57	213.47±32.96	53.80±7.78	77.48±9.95	0.75
218	10471-96-2	linalyl valerate	Ester	1510	12.46±0.41	4.57±0.37	4.32±0.32	5.84±0.31	4.90±0.50	0.77
219	141-16-2	citronellyl butyrate	Ester	1529	53.48±1.29	22.87±1.94	19.19±0.44	29.77±2.21	23.94±1.70	0.81
220	2407-43-4	5-ethyl-2(5H)-furanone	Lactone	966	274.24±12.58	264.61±5.04	267.47±3.53	209.10±6.45	198.37±2.14	1.09
221	502-44-3	ε -caprolactone	Lactone	1065	182.37±8.74	141.87±3.14	128.25±1.76	102.36±1.89	89.30±3.70	0.82
222	2381-87-5	dehydromevalonic lactone	Lactone	1169	69.29±7.79	1153.72±32.14	1017.59±14.74	894.01±13.38	813.42±42.21	0.98
223	698-10-2	abhexone	Lactone	1195	3870.41±158.31	2432.29±134.79	1711.82±33.62	918.37±36.01	723.44±53.28	0.83
224	698-76-0	δ -octalactone	Lactone	1288	5.11±0.44	2.58±0.15	3.02±0.18	1.95±0.13	3.58±0.15	0.97
225	39212-23-2	β -methyl-γ-octalactone	Lactone	1302	5.10±0.49	2.48±0.08	2.56±0.08	1.70±0.20	3.99±0.13	0.93
226	705-86-2	δ -decalactone	Lactone	1497	4.10±0.18	2.49±0.17	2.64±0.23	1.53±0.17	2.64±0.33	0.87
227	100-72-1	tetrahydro-2H-pyran-2-methanol	Oxygen heterocycle	992	22.40±1.61	27.40±0.78	22.35±0.32	35.81±2.57	84.46±1.85	1.01
228	92760-25-3	2,3-dehydro-1,8-cineole	Oxygen heterocycle	992	15.24±0.65	3.97±0.18	3.28±0.07	4.17±0.37	7.83±0.34	0.78
229	3777-69-3	2-pentylfuran	Oxygen heterocycle	993	160.73±6.60	43.86±1.02	40.80±0.77	50.69±2.62	156.03±1.88	0.96
230	1004-29-1	2-butyltetrahydro-guran	Oxygen heterocycle	1004	3.35±0.30	4.53±0.15	3.42±0.09	2.61±0.10	2.52±0.12	1.50
231	13360-64-0	2-ethyl-5-methylpyrazine	Oxygen heterocycle	1005	9.27±0.42	4.51±0.31	5.06±0.25	3.38±0.24	3.01±0.19	0.83
232	539-52-6	3-(4-methylpent-3-en-1-yl)furan	Oxygen heterocycle	1101	242.48±11.13	130.24±3.86	106.48±2.60	161.49±4.21	135.22±1.32	0.90
233	92356-06-4	rosefuran epoxide	Oxygen heterocycle	1176	91.46±4.09	65.06±2.15	53.56±1.11	48.15±0.97	49.62±2.08	0.80
234	3194-17-0	1-(furan-2-yl)pentan-1-one	Oxygen heterocycle	1176	19.91±1.03	13.69±0.66	11.93±0.42	14.51±0.46	16.94±0.82	0.82
235	1646-26-0	2-acetylbenzofuran	Oxygen heterocycle	1395	9.51±0.45	6.21±0.50	4.75±0.08	6.49±0.42	6.36±0.57	0.96
236	2445-82-1	3-methylcumarin	Oxygen heterocycle	1490	308.45±9.43	140.51±13.17	154.14±22.15	313.38±7.42	352.76±16.90	1.06
237	32737-14-7	2-ethoxy-3-methylpyrazine	Nitrogen heterocycle	1065	331.98±16.86	268.88±5.40	239.16±2.05	349.04±5.48	479.21±28.95	0.99

NO.	CAS	Compounds	Class	NIST_RI	FTL (ug/kg)	STL (ug/kg)	RTL (ug/kg)	SPTL (ug/kg)	DTL (ug/kg)	VIP value
238	1072-83-9	1-(1H-pyrrol-2-yl)ethan-1-one	Nitrogen heterocycle	1063	70.17±3.34	48.79±0.99	42.56±0.88	100.48±2.40	105.70±4.02	1.12
239	2314-78-5	1-ethylpyrrolidine-2,5-dione	Nitrogen heterocycle	1191	93.57±5.24	56.27±3.74	39.10±0.77	62.28±1.81	106.53±5.71	1.04
240	20189-42-8	methylethylmaleimide	Nitrogen heterocycle	1239	205.91±7.65	425.22±24.72	308.13±6.83	528.53±11.49	417.11±28.06	1.27
241	120-72-9	1H-indole	Nitrogen heterocycle	1292	137.63±3.86	68.34±8.64	30.75±1.04	22.54±1.85	27.96±2.42	0.86
242	59-48-3	2,3-dihydro-1H-indol-2-one	Nitrogen heterocycle	1487	159.94±4.83	61.59±5.97	64.45±8.72	21.38±2.12	27.48±3.19	0.75

Note: All values are shown as mean ± SD. FTL, fresh tea leaves; STL, steamed tea leaves; RTL, rolled tea leaves; SPTL, shaped tea leaves; DTL, dried tea leaves. VIP values, Variable Importance in the Projection.
