# Supplementary figures and images for: Analysis of the Changes in Volatile Components During the Processing of Enshi Yulu Tea
Source: Foods. 2024 Dec 9;13(23):3968. doi: 10.3390/foods13233968 (PMC11641255; doi:10.3390/foods13233968)

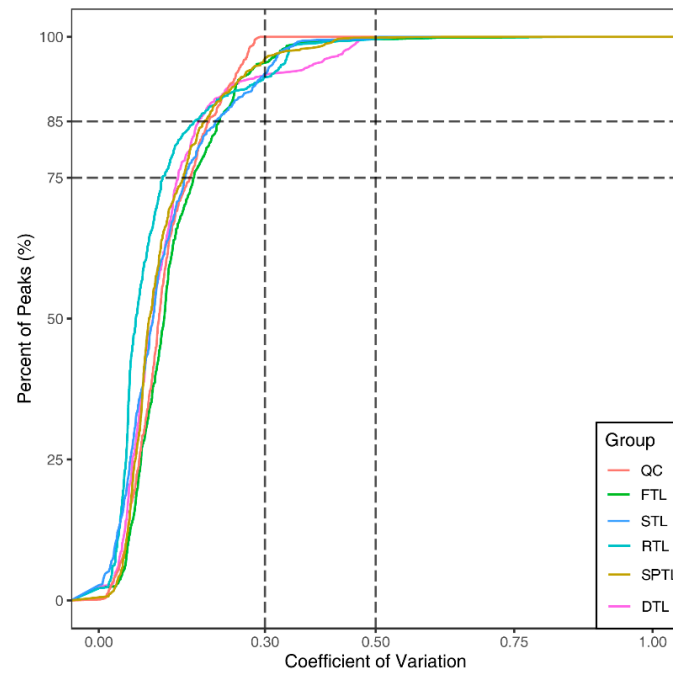

Figure S1. CV distribution of each group

Supplement: Supplementary file 1 [file foods-13-03968-s001.zip › Supplementary File-Figure S1.pdf]

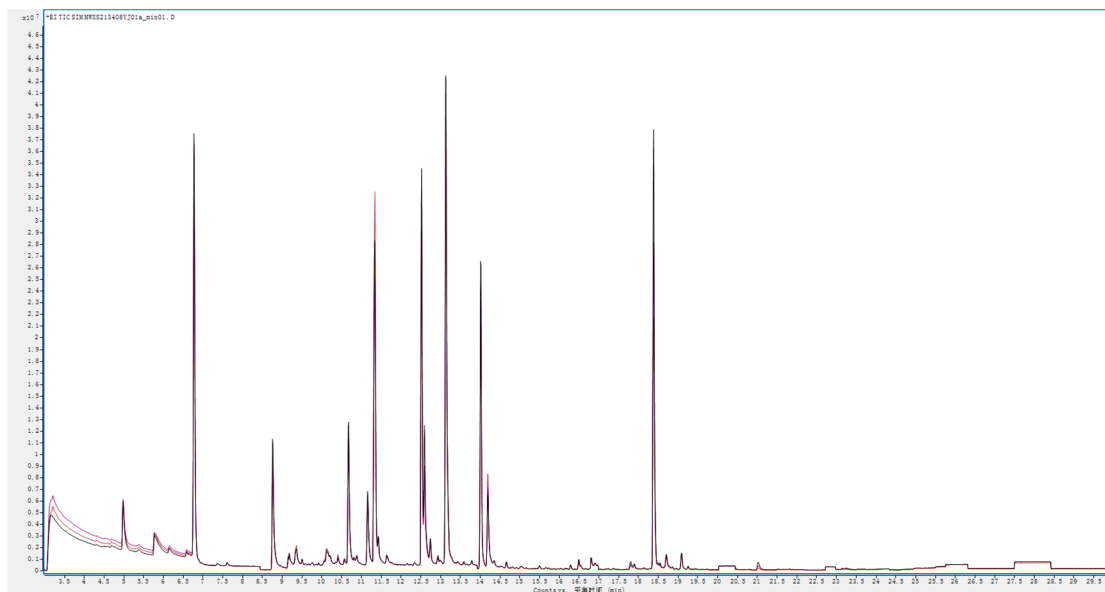

Figure S2. The essential spectrum detection of TIC overlap in the QC sample.

Supplement: Supplementary file 1 [file foods-13-03968-s001.zip › Supplementary File-Figure S2.pdf]
